# Subjective expectations regarding ageing: a cross-sectional online population survey in Hungary

**DOI:** 10.1007/s10198-019-01059-w

**Published:** 2019-05-20

**Authors:** Márta Péntek, Ottó Hajdu, Fanni Rencz, Zsuzsanna Beretzky, Valentin Brodszky, Petra Baji, Zsombor Zrubka, Klára Major, László Gulácsi

**Affiliations:** 1 Department of Health Economics, Corvinus University of Budapest, Fővám tér 8., Budapest, 1093 Hungary; 2 Department of Comparative Economics, Institute of Business Economics, Eötvös Loránd University, Szép u. 2, Budapest, 1053 Hungary; 3 Premium Postdoctoral Research Programme, Hungarian Academy of Sciences, Nádor u. 7, Budapest, 1051 Hungary; 4 Doctoral School of Business and Management, Corvinus University of Budapest, Fővám tér 8., Budapest, 1093 Hungary; 5 Department of Macroeconomics, Corvinus University of Budapest, Fővám tér 8., Budapest, 1093 Hungary

**Keywords:** Subjective expectations, Ageing, EQ-5D-5L, Happiness, Employment, Informal care, I19

## Abstract

**Background:**

We aimed to investigate individuals’ subjective expectations regarding health and happiness alongside their provisions on life circumstances for older ages.

**Methods:**

A cross-sectional online survey was performed involving a representative sample (*N* = 1000; mean age 50.9, SD = 15.4; female 54.5%) in Hungary. Subjective expectations on health status (EQ-5D-3L/-5L, GALI, WHO-5), happiness (0–10 VAS), employment status, care time, and forms of care for ages 60, 70, 80, and 90 were surveyed.

**Results:**

Current mean EQ-5D-5L was 0.869 (SD = 0.164) and happiness was 6.7 (SD = 2.4). Subjective life expectancy was 80.9 (SD = 11.1), and median expected retirement age was 65. Mean expected EQ-5D-5L for ages 60/70/80/90 was 0.761/0.684/0.554/0.402, and no activity limitations (GALI) were expected by 64%/40%/18%/14%, respectively. Expected happiness score was 6.8/6.7/6.2/5.7, and a decrease in mental well-being (WHO-5) was provisioned. A substantial increase in drug expenses and care time was anticipated, but only 52% thought to have extra income besides pension. The great majority expected to be helped by the family (77%/72%/53%/40%) if needed. Educational level, GALI, and longevity expectations were significant predictors of EQ-5D-5L expectations using a standard 5% significance level of decision. Current happiness was major determinant of expected future happiness.

**Conclusions:**

Individuals expect a significant deterioration of health with age but only a moderate decrease in happiness. Overestimation of future activity limitations suggests a gap between statistical and subjective healthy life expectancy. The majority expects to rely on informal care in the elderly. Raise in retirement age is underestimated. Our results can be used as inputs for economic modelling of labor force participation and ageing.

**Electronic supplementary material:**

The online version of this article (10.1007/s10198-019-01059-w) contains supplementary material, which is available to authorized users.

## Introduction

Assessing individuals’ subjective expectations has come into focus in the past years in healthcare. Patient-reported outcomes (PRO) play an increasing role in drug registration process and in medical decision making, as these are used in clinical trials and in everyday clinical practice to assess disease severity and therapeutic effectiveness [1, 2]. Given that PROs reflect subjective perceptions of the patients, several personal factors might influence the valuation of these outcomes, such as preferences for some treatment forms or acceptability of certain health problems. Subjective expectations regarding health can be deterministic in the self-perceived health status assessment and unrealistic expectations about future health may lead to distortion in the evaluation of health gains [3, 4]. Inaccurate health expectations may have influence also on actual health behaviour in terms of, for instance, participation in preventive care, living a healthy lifestyle or choosing an insurance plan.

Brouwer and colleagues highlighted that overestimation may be present in life expectancy and age, current health, and perception of healthy lifestyle are important explanatory factors of people’s subjective expectations regarding future health-related quality of life [3, 4]. Similar findings were reported from Hungary in a study involving a convenient online sample of the general population [5]. Underestimation of future health status from age 70 and onwards (i.e., expecting worse states than the general population has in these ages) was typical in both countries. Subsequent studies involving patients with chronic diseases (rheumatoid arthritis, psoriasis, and age-related macular degeneration) in Hungary revealed that patients’ do expect a significant deterioration of health with age but may overestimate treatment effects on the short term [5–7]. However, up to date, little is known about how individuals think about the consequences of age-related worsening of health, namely, how they plan to cope with health problems and how it will affect their happiness and mental well-being. Subjective expectations regarding retirement, needs, and demands for care services and financial consequences are poorly studied, although these are highly relevant for workforce, healthcare, and social care planning in ageing societies [8, 9].

From the methodological point of view, several questionnaires have been developed in the past years to measure patients’ expectations regarding healthcare services, treatment choices, or outcomes [10–16]. However, these are mainly disease-specific or focus on specific needs and hence cannot be used in studies of the general population. Brouwer and colleagues were the first to use the EQ-5D instrument, a preference-based health status measure, to assess subjective health expectations (SHE) for future ages [3, 17]. Applying the EQ-5D to explore SHE has important advantages. First, population norm data are available in many countries with this general health status measurement tool [18]. Comparing EQ-5D-based SHE to the EQ-5D health status of the age-matched general population allows to identify tendencies of over- and underestimation of future health. Second, EQ-5D has been used in routine outcomes measurement in healthcare systems over the past years [19]. Getting an insight into individuals’ and patients’ SHE with the EQ-5D can help a better understanding and interpretation of healthcare performance results. Third, EQ-5D is the most often used tool to calculate quality-adjusted life years (QALY) in economic evaluations [20]. EQ-5D-based SHE assessments can provide, therefore, comparable data on individuals’ subjective QALY expectations [4]. In the previous SHE studies, the three-level version of the EQ-5D questionnaire (EQ-5D-3L) was used to assess health provisions for future ages [3–7]. A new version with five response levels (EQ-5D-5L) was developed in 2009 to improve sensitivity and reduce ceiling effects, as compared to the EQ-5D-3L [21]. Population norm and valuation studies with the 5L have been performed and are ongoing in various countries and an increasing spread of its use can be observed [18, 22]. Therefore, we believe that there will be an increasing need for SHE data with the EQ-5D-5L and also to find ways to convert 3L- and 5L-based SHE results to each other.

While EQ-5D is widely used in health economic evaluations, large international social surveys such as the European Statistics of Income and Living Condition (EU-SILC) or the European Health Interview Survey (EHIS) apply the Minimum European Health Module including the Global Activity Limitations Indicator (GALI) [23]. GALI is the measure underlying the European indicator Healthy Life Years (HLY) hence using GALI to assess SHE can provide valuable information about potential discrepancies between statistically and subjectively expected HLYs [24].

The primary aim of our study was, therefore, to investigate subjective expectations regarding health and happiness at future ages and analyze the determining factors. To provide comparable data with clinical studies and routinely collected health statistics, we used four standardized health status measures (EQ-5D-3L, EQ-5D-5L, GALI, and WHO-5) to explore SHE. Secondarily, we aimed to investigate the expected drug costs, care needs and forms of care for older ages, as well as the expected retirement age and financial circumstances for the elderly.

## Methods

### Sample and study design

A cross-sectional online survey was performed among the adult Hungarian general population in early 2019. Authorization by the national ethical committee was obtained (5113-2/2018/EKU). Respondents gave their written consent to participate in the study. Data collection was preceded by a pilot survey (*N* = 204) before the questionnaire was finalized. Programing the questionnaire to online and recruitment of the respondents was conducted by a survey company (Big Data Scientist Kft.). We aimed to achieve a sample (*N* = 1000) representative for the Hungarian adult population in terms of gender, age (by age groups of 18–24, 25–34, 35–44, 45–54, 55–64, and a reasonable sample for age 65 and over), educational level (primary, secondary, and tertiary) and type of settlement (capital, town, and village), and region (Central, Eastern, and Western) of the country using quotas.

### Measurement tools

#### EQ-5D-5L and EQ-5D-3L

The EQ-5D-5L questionnaire is a health status measure that consists of two parts, a descriptive system and an EQ VAS. The descriptive system focuses on five dimensions of current health: mobility, self-care, usual activities, pain/discomfort, and anxiety/depression with five response categories for each (no problems—1, slight problem—2, moderate problems—3, severe problem—4, and extreme problems—5) [21]. To each health state description, a utility score (EQ-5D-5L index) can be attached reflecting the general population’s preference for the given health state. In the absence of a Hungarian value set, we used the tariffs of England [25, 26]. The second part of the questionnaire is a visual analogue scale (EQ VAS) ranging from 0 to 100 representing the worst and best imaginable health state the respondent can imagine.

The EQ-5D-3L is the original instrument with three response levels (1—no problems; 2—some/moderate problems; and 3—unable/extreme problem) in the descriptive system [17]. We used the UK value set to calculate EQ-5D-3L index scores [27].

#### Minimum European Health Module (MEHM)

The MEHM consists of three general questions characterizing three different concepts of health: self-perceived health, chronic morbidity, and activity limitation. Self-perceived health covers the self-assessment of a person’s own health in general, with answer categories: “Very good/Good/Fair/Bad/Very bad”, while chronic morbidity focuses on the presence of any long-standing (for at least the past 6 months) health problem (yes/no). Global Activity Limitation Indicator (GALI) refers to the presence of long-standing (for at least the past 6 months) participation limitation due to health problems (severely limited/limited but not severely or/not limited at all) in activities that people usually do.

#### Happiness

Happiness was measured on 11-step numeral happiness scale (hereinafter happiness VAS) in which the extremes were denoted as “completely unhappy” (0) and “completely happy” (10). Respondents were asked to indicate their current happiness by marking a point between 0 and 10 on the scale. This tool is also part of the CarerQol questionnaire assessing the quality of life of informal caregivers [28].

#### World Health Organisation–Five Well-Being Index (WHO-5)

The WHO-5 is a self-reported measure of mental well-being. This questionnaire consists of five items (feel cheerful and in good spirits; calm and relaxed; active and vigorous; wake up fresh and rested; and daily life filled with thing that interests me) and a six-point Likert scale for each (all of the time—5, most of the time—4, more than half of the time—3, less than half of the time—2, some of the time—1, and at no time—0) with respect to the past 2 weeks. The score is calculated by totaling the figures of the five answers (range 0–25). In our study, we used a modified version of the questionnaire with a simplified five-point Likert-scale (‘some of the time’ was left out) to make the expectations questions more simple. Similar modification of the questionnaire has already been used in population surveys [29, 30]. For the analysis, responses were grouped into two subgroups (all/most of the time; other) and no score was calculated.

### Assessment of respondents’ current status

Basic demographic data of the respondents were recorded. Age of the respondents’ youngest and oldest child (if any) were registered. The number of persons living in the household, as well as the monthly net income and spending on drugs was surveyed.

We used the validated Hungarian version of the EQ-5D-5L questionnaire and the MEHM to assess the respondents’ health status, as well as the happiness VAS and the simplified WHO-5. Body mass index (BMI) and self-perceived healthy lifestyle were recorded. Smoking habits, sport activities, and alcohol consumption were assessed using the same questions as the European Health Interview Survey (EHIS) [31].

Current drug expenditures were assessed on the household level. Informal care received (help from family members to perform everyday activities that are limited due to health problems or ageing) was surveyed and respondents were asked whether they themselves were informal caregivers.

### Assessment of expectations regarding future health and happiness

In the second part of the questionnaire, participants were asked to indicate the health status they expect for future ages. The questions were focused on ages 60, 70, 80, and 90, and only those respondents were required to answer who were younger than the age the question was referring to. Questions were formulated based on the descriptive part of the EQ-5D-5L and the EQ-5D-3L, GALI, happiness VAS, and simplified WHO-5 (Supplementary Table S1).

### Assessment of subjective perspectives on drug costs, care needs, and access to care

We asked the participants to indicate the expected spending on pharmaceutical products in their household at ages 60, 70, 80, and 90, in the percentage of household’s monthly net income. Estimates on future care needs (h/week) and type of care they will use were surveyed (staying in their own home and being helped by volunteer family members/non-professionals paid by the respondent; moving to the home of close relatives to be helped; moving to institutions provided by the healthcare/social system with no co-payment; moving to institutions by own choice with significant costs; and other).

### Assessment of expected retirement age and financial circumstances for the elderly

To access the expected labor and financial situation in the future, subjective life expectancy was asked and the age respondents expect to do paid work. We surveyed also whether they think to have any regular income other than their pension after having stopped working (income from property or assets; private insurance or financial aid from family member; other).

### Statistical analysis

Survey data were recorded in a database created in IBM SPSS Statistics 25 (IBM SPSS, Version 25.0. Armonk, NY, USA: IBM Corp., 2012). Descriptive statistics were conducted and explanatory factors associated with subjective health and happiness expectations were investigated. To analyze the determinants of subjective expectations on future health (measured with the EQ-5D-5L index) and future happiness (happiness VAS), we built separate regression models. The explanatory variables in our models were: socio-demographic characteristics (gender, marital status, level of education, employment, and household net income); measures of current health and happiness (EQ-5D-5L index, EQ VAS, self-perceived health scale, GALI, chronic morbidity, and happiness VAS); determinants of health (self-perceived healthy lifestyle, BMI, alcohol and tobacco consumption, and sport activities); informal care experience (informal care received or provided); and longevity factors (close relatives’ age at death and subjective life expectancy). Categorical (nominal) variables (gender, employment, and marital status) were included in the model using dummy variables. Modelling the marginal effects of explanatory variables on the EQ-5D-5L dependent variable was estimated by linear regression coefficients yielding also the standardized version of the coefficients, significance *p* values, and goodness-of-fit *R*^2^ indices. The regression models for current and expected happiness were built similarly. While modelling statistical correlations and regression relationships, the computations were carried out by the means of stepwise ordinary least squares (OLS) method. As a result of OLS, we get the rank, significance and explanatory power of independent variables that have entered the current model. The subset of the relevant predictors has been selected based on the adjusted *R*^2^ goodness-of-fit measure. In addition, the so-called standardized regression coefficients represent the relative importance of the explanatory variables. The applied currency exchange rate was: €1 = 314 HUF.

## Results

### Sample

Our sample consisted of 1000 respondents (Table 1). Descriptive statistics show (using a conventional 5% significant level of decision) that the sample represents the whole population considering basic demographic distributions.

**Table 1:** Sociodemographic characteristics of the sample and general population reference values

Variable	Category	Sample, % (*N* = 1000)	Sample (*N* = 1000) weighted for gender, age, educational level, region and type of settlement^a^ (%)
Gender	Female	54.5	53.4
	Male	45.5	46.6
Age (years)	18–24	5.2	10.6
	25–34	11.5	16.9
	35–44	22.1	18.8
	45–54	18.0	15.5
	55–64	19.2	17.6
	65+	24.0	20.6
Educational level	Primary	30.0	51.0
	Secondary	42.2	31.3
	High school/university	27.8	17.7
Region	Central	33.8	30.0
	Eastern	38.2	39.6
	Western	28.0	30.4
Type of settlement	Capital (Budapest)	22.3	18.1
	Town	52.3	51.9
	Village	25.4	30.0
Marital status	Married	42.5	38.9
	Living together	17.1	20.1
	Single	20.1	22.5
	Divorced	11.6	10.1
	Widow	7.5	6.7
	Other	1.2	1.6
Employment status	Full time	47.0	43.9
	Part-time	4.6	6.0
	Retired	29.5	25.7
	Disability pensioner	4.2	5.0
	Student	1.6	2.9
	Unemployed (seeking job)	4.7	5.8
	Unemployed (not seeking job)	1.0	0.9
	Housewife/husband	3.2	4.6
	Other	4.2	5.2
Household net income, Euro/month^b^	0–159	2.6	3.1
	160–318	4.5	6.1
	319–478	9.9	11.3
	479–637	12.7	12.6
	638–796	12.3	12.7
	797–955	13.1	12.8
	956–1115	8.3	8.0
	1116–1274	5.5	5.3
	1275–1433	3.4	2.6
	1434–1592	4.6	3.7
	1593–	5.8	4.9
	Missing data	17.3	17.0

^a^Based on general population over 18 years of age, 2011 European Census Data. For all analyses, we used the data of the non-weighted sample

^b^Conversion: 1 EUR = 314 HUF

The rate of females was 54.5% and average age was 50.9 (15.4) years (range 18–85). Based on respondents’ age, questions about subjective expectations for future ages of ≥ 60, ≥ 70, ≥ 80, and 90 were relevant for 623, 898, 990, and 1000 respondents, respectively. Main socio-demographic characteristics of the participants are presented in Table 1. The average household size was 2.5 (SD = 1.7), 23.8% were one-person households, and in 76.3% of the cases, no child (aged < 18) lived in the household. These results are in line with Hungarian micro-census data from 2016 [32]. The majority (69.8%) had children (of any age); among them, 34.1% had only one child (with mean age of 24.5, SD = 15.8 years). Age of the youngest and oldest child among respondents having more than one child was 26.6 (SD = 14.0, range 0–61) and 32.7 (SD = 13.4, range: 0-65), respectively.

### Current health, happiness, drug expenditures, and informal care

Current status of the sample is presented in Tables 2 and 3. EQ VAS result of the sample was similar to that of the general population (mean 76.2 vs. 71.1) [18, 33] (EQ-5D-5L index population norm is not yet available in Hungary.). Altogether, 57.5% of the sample perceived to be in very good or good health status which is very close to the 60% in Eurostat statistics (aged 16 and over), but more respondents reported to have a chronic morbidity (52.5% vs. 38.2%) [34, 35].

**Table 2:** Health status and some determinants of health (*N* = 1000)

Variable	Category	*N* (%)
Self-perceived health^a^	Very good	114 (11.4%)
	Good	461 (46.1%)
	Fair	327 (32.7%)
	Bad	87 (8.7%)
	Very bad	11 (1.1%)
Chronic morbidity	Yes	525 (52.5%)
	No	380 (38.0%)
	I do not know	86 (8.6%)
	I do not want to answer	9 (9%)
Body mass index (BMI) kg/m^2^	Underweight (< 18.5)	36 (3.6%)
	Normal (≥ 18.5 and < 25)	309 (30.9%)
	Overweight (≥ 25 and < 30)	345 (34.5%)
	Obese (≥ 30)	310 (31.0%)
Healthy lifestyle	Healthier than most others	210 (21.0%)
	Comparable to others	614 (61.4%)
	Less healthy than most others	176 (17.6%)
Smoking status	Current smoker	299 (29.9%)
	Quitted smoking within a year	25 (2.5%)
	Quitted smoking more than a year ago	252 (25.2%)
	Never smoked	424 (42.4%)
Alcohol consumption	Every day or nearly every day	67 (6.7%)
	1–6 days per week	205 (20.5%)
	1–3 times per month	217 (21.7%)
	Less than once per month	248 (24.8%)
	Not in the past 12 months	76 (7.6%)
	Never or only a few times in life	187 (18.7%)
Sports (min. 10 min’ sport at least one day per week)	Yes	515 (51.5%)
	No	485 (48.5%)

**Table 3:** Current and expected status for future ages

Variable	Current	Subjective expectations for age …			
		60	70	80	90
*N*	1000	623	898	990	1000
EQ-5D-5L index (− 0.285 to 1.000), Mean (SD)	0.869 (0.164)	0.761 (0.263)	0.684 (0.306)	0.554 (0.370)	0.402 (0.423)
EQ-5D-3L index (− 0.594 to 1.000), Mean (SD)	NA	0.712 (0.356)	0.569 (0.429)	0.333 (0.522)	0.113 (0.571)
Happiness (0–10), Mean (SD)	6.7 (2.4)	6.8 (2.5)	6.7 (2.5)	6.2 (2.7)	5.7 (3.1)
Activity limitation (GALI)^a^, *N* (%)					
Not limited at all	619 (61.9%)	396 (63.5%)	358 (39.9%)	182 (18.4%)	136 (13.6%)
Limited but not severely	310 (31.0%)	202 (32.4%)	453 (50.4%)	556 (56.2%)	411 (41.1%)
Severely limited	46 (4.6%)	25 (4.0%)	87 (9.7%)	252 (25.5%)	453 (45.3%)
WHO-5, *N* (%)					
Feel cheerful and in good spirits—all or most of the time	522 (52.2%)	364 (58.4%)	460 (51.2%)	399 (40.3%)	326 (32.6%)
Less or never	478 (47.8%)	259 (41.6%)	438 (48.8%)	591 (59.7%)	674 (67.4%)
Feel calm and relaxed—all or most of the time	439 (43.9%)	312 (50.1%)	436 (48.6%)	386 (39.0%)	319 (31.9%)
Less or never	561 (56.1%)	311 (49.9%)	462 (51.4%)	604 (61.0%)	681 (68.1%)
Feel active and vigorous	457 (45.7%)	272 (43.7%)	342 (38.1%)	289 (29.2%)	224 (22.4%)
Less or never	543 (54.3%)	351 (56.3%)	556 (61.9%)	701 (70.8%)	776 (77.6%)
Wake up fresh and rested	356 (35.6%)	242 (38.8%)	339 (37.8%)	291 (29.4%)	245 (24.5%)
Less or never	644 (64.4%)	381 (61.2%)	559 (62.2%)	699 (70.6%)	755 (75.5%)
Daily life filled with thing that interests me	366 (36.6%)	252 (40.4%)	334 (37.2%)	292 (29.5%)	245 (24.5%)
Less or never	634 (63.4%)	371 (59.6%)	564 (62.8%)	698 (70.5%)	755 (75.5%)
Care need^b^ (h/week), *N* (%)					
0	917 (91.7%)	436 (70.0%)	409 (45.5%)	192 (19.4%)	126 (12.6%)
1–4	23 (2.3%)	112 (18.0%)	270 (30.1%)	303 (30.6%)	200 (20.0%)
5–7	10 (1.0%)	38 (6.1%)	123 (13.7%)	242 (24.4%)	175 (17.5%)
8–28	36 (3.6%)	21 (3.4%)	55 (6.1%)	142 (14.3%)	213 (21.3%)
29–56	10 (1.0%)	10 (1.6%)	25 (2.8%)	65 (6.6%)	146 (14.6%)
More than 56	4 (0.4%)	6 (1.0%)	16 (1.8%)	46 (4.6%)	140 (14.0%)
Expects to be in a paid job (part time or full time)^c^					
Yes	NA	540 (92.8%)	239 (35.5%)	45 (6.5%)	20 (2.9%)
No	NA	42 (7.2%)	433 (64.4%)	643 (93.5%)	668 (97.1%)

*NA* not available/not applicable

^a^Current status on GALI, further responses: ‘I do not know’ *N* = 19 (1.9%); ‘I do not want to answer’ *N* = 6 (0.6%)

^b^For the current status, we present the hours of care received (professional and informal care together)

^c^Those respondents who have already definitely stopped working were not included in this analysis

The happiness score of the sample was in average 6.7 (SD = 2.4; range 0–10). For comparison, in the European Quality of Life Survey (EQLS), the average happiness of the Hungarian adult population was 7.0 on a scale between 1 and 10 [36].

The average monthly spending on drugs in the household was €29.3/month (SD = 43.5) and an increase was observed across age groups (55–64, 65–74, and 75–84: mean €28.7, €38.1, and €47.6, respectively). In Hungary, the yearly drug expenses per capita in average were €86 in 2010 and €115 in 2017, and this latter corresponds to about €22/month per household in 2017 (2.9% of the average household income) [37, 38].

Altogether, 76 respondents reported to receive informal care and 11 paid care. Further 30 individuals did not receive care, but would have needed according to their self-report. Altogether, 11% of the sample was caregiver (for at least 6 weeks) at the time of the survey and the average care time was 54.4 (15.7) h per week. Further 27.9% provided informal care in the past 10 years (but not now). In age-group 50 and over (*N* = 542), the number of current informal caregivers was 73 (13.5%) that is close to the OECD average (13.3%) [39].

### Expected health and happiness

Participants’ subjective expectations for future ages are presented in Table 3. There was only one respondent in age group 85+; hence, we got no accountable data to make comparisons between the expectations for age 90 and the sample’s age-matched actual score.

**Fig. 1:**
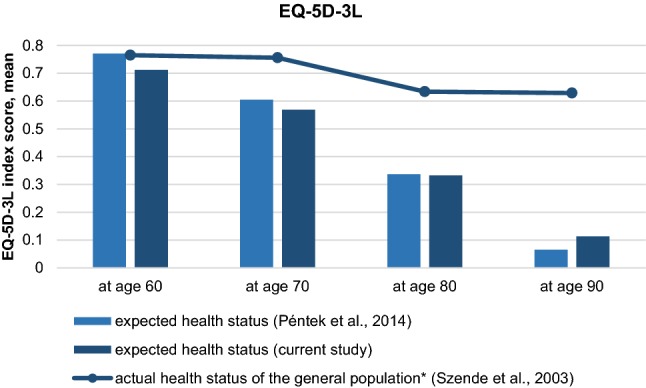
Expected health status for future ages (current and a previous study) in comparison with the health status of the general population. *in age groups 55–64, 65–74, 75–84, and 85+

The expected mean EQ-5D-5L scores for ages 60, 70, and 80 were lower than of the actual EQ-5D-5L score of the age-matched sample (in age groups 55–64, 65–74, and 75–84: 0.761 vs. 0.853; 0.684 vs. 0.840; and 0.554 vs. 0.820, respectively). Respondents expected a sharp deterioration of health with age as measured by the EQ-5D-3L and the difference is remarkable compared to the Hungarian general population’s health status from age 70 and over (Fig. 1).

Participants expected in average a decline of happiness with age (Table 3). According to EQLS data, in Hungary, happiness (on a 1–10 scale) was the highest in age group 25–34 (7.7) with a decreasing tendency with age, being the lowest (6.3) in age group 65+, that is close to the expected scores of the sample [36].

Correlations between expected EQ-5D-3L, EQ-5D-5L, and happiness VAS were positive and significant for all the four future ages. It was high (Spearman rank correlation coefficients > 0.7) between the two versions of the EQ-5D and low bordering to moderate (Spearman rank correlation coefficients around 0.4 and 0.5) between the expected EQ-5D measures and expected happiness VAS (data not shown.)

### Determinants of subjective expectations on future health (EQ-5D-5L) and happiness

In the models for SHE (EQ-5D-5L index) for future ages, current EQ-5D-5L index was significant for expected status at ages 60 and 70 (Table 4). For ages 70, 80, and 90, current activity limitation (GALI) and educational level were significant predictors. BMI was negatively associated with SHE for ages 70 and 80, while subjective life expectancy was positively associated for ages 80 and 90.

**Table 4:** Determinants of current and expected health for future ages

Predictor variables	Dependent variable: EQ-5D-5L index		Dependent variable: expected EQ-5D-5L index at age 60		Dependent variable: expected EQ-5D-5L index at age 70		Dependent variable: expected EQ-5D-5L index at age 80		Dependent variable: expected EQ-5D-5L index at age 90	
	*R*^2^ 0.760		*R*^2^ 0.881		*R*^2^ 0.735		*R*^2^ 0.730		*R*^2^ 0.640	
	Unstandardized coefficients B	Standardized coefficients Beta	Unstandardized coefficients B	Standardized coefficients Beta	Unstandardized coefficients B	Standardized coefficients Beta	Unstandardized coefficients B	Standardized coefficients Beta	Unstandardized coefficients B	Standardized coefficients Beta
(Constant)	0.465*^(0)^	…	0.388*^(0)^	…	− 0.795*^(0)^	…	− 0.664^(0)^	…	− 1.771***^(0)^	…
EQ-5D-5L index	…	…	1.187***^(1)^	0.922	0.870***^(1)^	0.548	0.326^(1)^	0.194	…	…
EQ VAS	0.003**^(6)^	0.292	…	…	…	…	0.004**^(5)^	0.237	0.004^(1)^	0.219
Activity limitation (GALI)	0.085**^(4)^	0.233	…	…	0.214**^(7)^	0.355	0.153**^(9)^	0.248	0.310***^(5)^	0.488
Self-perceived health	− 0.062*^(1)^	− 0.244	…	…	…	…	…	…	0.088^(16)^	0.201
Chronic morbidity	…	…	…	…	− 0.262^(6)^	− 0.133	…	…	…	…
Current happiness	0.015*^(8)^	0.159	…	…	− 0.029^(12)^	− 0.188	…	…	…	…
Age	0.002^(2)^	0.126	− 0.008**^(4)^	− 0.214	…	…	…	…	…	…
Gender	…	…	− 0.539***^(2)^	− 0.656	− 0.096^(3)^	− 0.118	− 0.189***^(2)^	− 0.242	− 0.119^(4)^	− 0.148
Educational level	− 0.015^(13)^	− 0.093	…	…	0.091***^(2)^	0.307	0.052**^(3)^	0.189	0.073**^(2)^	0.254
Subjective life expectancy	0.003***^(3)^	0.229	…	…	0.002^(13)^	0.112	0.005**^(4)^	0.200	0.010***^(3)^	0.380
Kins’ age at death	…	…	…	…	…	…	…	…	…	…
Body Mass Index category	− 0.047**^(5)^	− 0.183	…	…	− 0.201***^(5)^	− 0.467	− 0.137***^(10)^	− 0.321	− 0.078^(12)^	− 0.178
Informal care received (h/week)	…	…	…	…	…	…	…	…	− 0.003^(15)^	− 0.147
Informal caregiver	…	…	0.320***^(3)^	0.399	…	…	…	…	− 0.084^(17)^	− 0.103
Marital status: single	− 0.082^(7)^	− 0.118	…	…	…	…	…	…	− 0.173^(10)^	− 0.144
Marital status: divorced	…	…	…	…	…	…	…	…	…	…
Marital status: widow	− 0.055^(14)^	− 0.093	…	…	…	…	…	…	…	…
Marital status: has a partner	− 0.070^(15)^	− 0.081	…	…	…	…	− 0.165^(15)^	− 0.115	…	…
Other marital status	…	…	…	…	…	…	…	…	…	…
Net household monthly income	…	…	…	…	− 0.034^(8)^	− 0.198	…	…	…	…
Self-perceived healthy lifestyle	…	…	…	…	0.176*^(11)^	0.267	0.113*^(12)^	0.188	0.113^(13)^	0.182
Smoking status	− 0.013^(16)^	− 0.065	…	…	0.137***^(4)^	0.415	0.061*^(11)^	0.185	0.061^(9)^	0.178
Sport activities	− 0.024^(10)^	− 0.052	…	…	…	…	− 0.074^(7)^	− 0.094	− 0.115^(8)^	− 0.142
Alcohol consumption	− 0.007^(11)^	− 0.069	…	…	…	…	− 0.015^(6)^	− 0.091	0.016^(18)^	0.092
Employment status: works part-time	0.143^(12)^	0.077	…	…	…	…	0.319^(13)^	0.103	…	…
Employment status: retired	…	…	…	…	…	…	…	…	− 0.196^(14)^	− 0.242
Employment status: disability pensioner	…	…	…	…	…	…	− 0.073^(8)^	− 0.076	− 0.269*^(7)^	− 0.271
Employment status: student	…	…	…	…	…	…	…	…	…	…
Employment status: unemployed (seeking job)	− 0.158^(9)^	− 0.120	0.466**^(5)^	0.237	− 0.182^(9)^	− 0.092	…	…	0.431^(6)^	0.188
Employment status: unemployed (not seeking job)	…	…	…	…	0.601*^(10)^	0.217	0.386^(14)^	0.124	…	…
Employment status: housewife/husband	…	…	…	…	…	…	…	…	…	…
Other employment status	…	…	…	…	…	…	…	…	0.205^(11)^	0.109

Significance level (*p* value): *between 0.1 and 0.05; **between 0.05 and 0.01; *** < 0.01; No*: *p* value > 0.1

(#)Stepwise entry number

Explanatory variables indicated as ‘…’ in the table were not involved into the model by the regression model specification. For dummies (marital and employment status): the first category (marital status: married; employment status: works in a full-time job) was used as reference. Maximum points of corrected *R*^2^ define the actual list of predictors

Coding: GALI: 1—severely limited, 2—limited but not severely, and 3—not limited; gender: 0—female and 1—male; educational level: 1—no primary education, …, 10—university; chronic morbidity: 1—yes and 2—no; Kins’ age at death: 1—55–64 years, 2—65–74, 3—75–84, 4—85–94, 5—95 years, or over; body mass index category: 1—underweight, 2—normal, 3—overweight, and 4—obese; self-perceived health: 1—very good, 2—good, 3—fair, 4—bad, and 5—very bad; informal caregiver experience: 1—yes, currently, 2—yes in the past but not currently, and 3—no; net household monthly income: 1—0–159 EUR/month, …, 11—1593 EUR/month or over; self-perceived healthy lifestyle: 1—healthier than others, 2—comparable to others, and 3—less healthy than others; smoking status: 1—current smoker, 2—quitted smoking within a year, 3—quitted smoking more than a year ago, and 4—never smoked; alcohol consumption: 1: every day or nearly every day, …, 9: never only a few times in my life; dummy: 1—yes, 2—no

Current happiness was significant explanatory variable for expected happiness for all the four future ages (Supplementary Table S2). Current self-perceived health and informal care received (h/week) were significant predictors of future happiness at ages 70, 80, and 90. Gender was significant in the model for expected happiness at ages 70 and 80. The presence of chronic morbidity was negatively associated with expected happiness for ages 70 and 80. Current informal care received (h/week) was positively associated with happiness expectations for ages 70, 80, and 90. Actual monthly income was not significant in any of the models. Only few of the variables expressing marital and employment status were significant in the models.

### Provisions on drug costs, care needs, and forms of care for future ages

**Fig. 2:**
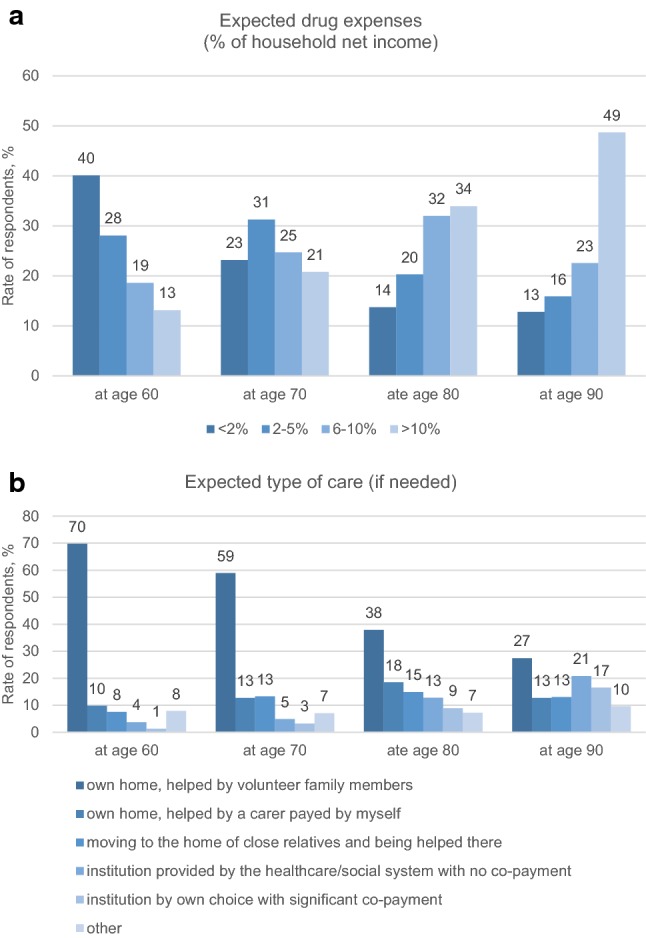
Expected drug expenses and forms of care for future ages

Drug expenses and types of care expected for ages 60, 70, 80, and 90 are resented by Fig. 2. A shift in direction of a higher share of drug expenses of the household net income was forecasted by the participants (Fig. 2a), and an increasing need for care (h/week) was provisioned (Table 3). Expectations on being helped in their own home by family members (informal care) were dominant for all future ages, although with a decreasing tendency (Fig. 2b). Characteristics of the sample expecting different forms of care at age 80 are presented in supplementary file (Supplementary Table S3).

### Expected working status and incomes for future ages

A substantial number of respondents (*N* = 40) indicated ages between 101 and 120 for subjective life expectancy. Applying a maximum limit of 100 years, respondents (*N* = 941) expected to live up to age 80.9 (SD = 11.1), females 81.1 (SD = 11.5), males 80.7 (SD = 10.8). For comparison, life expectancy at birth was 79.0 years for females and 72.4 for males in 2017 in Hungary and life expectancy at age 65 was 14.6 and 18.7 years, respectively.

Share of respondents who expected to work in a paid job at ages 60, 70, 80, and 90 is presented in Table 3 (those who have definitely stopped working in a paid job were excluded from the analysis). Median expected age of stopping doing paid work was 65 years, and it was mean 63.5 (SD = 7.5), 66.1 (SD = 7.3), 67.2 (SD = 8.1), 67.2 (SD = 7.5), 66.8 (SD = 5.6), and 75.7 (SD = 7.0) in age groups 18–24, 25–34, 35–44, 45–54, 55–64, and 65+, respectively. Women expected somewhat lower age than men (mean 66.0 vs. 68.5), and altogether, 48.4% expected not to have any extra income besides pension, respectively. Private insurance (22.2%), renting out the house (14.8%), financial help from family members (10.3%), and stocks (6.3%) were marked and a large variety of other possibilities were mentioned (e.g., selling the flat, from savings, etc.) but all at low proportions.

## Discussion

In this study, we assessed subjective expectations regarding future health and happiness of the Hungarian population in an online, cross-sectional population-based survey and analyzed their determinants. According to our best knowledge, this is the first study to investigate subjective happiness expectations alongside SHE for the elderly. Further novelty of this research is that we used different measurement tools in parallel to assess SHE. We applied both versions (3L and 5L) of the EQ-5D questionnaire to explore SHE, allowing comparisons with 3L- and 5L-based studies. We used the GALI instrument to provide comparable data for healthy life year (HLY) calculations and the WHO-5 questionnaire to obtain information about mental well-being expectations. Moreover, subjective expectations on drug expenses, care needs, and forms of care for the elderly were surveyed, and provisions on working and financial status for older ages were investigated aiming to provide a broader picture about the population’s ideas regarding ageing.

Our results on SHE as measured by the EQ-5D-3L confirm the findings of the previous studies in The Netherlands and of a non-representative population survey in Hungary [3–5]. Individuals expect a deterioration of health with age and an underestimation of health can be observed for age 70 and over compared to the age-matched actual health status of the population (Fig. 1). Adaptation to deteriorating health with age might be one explanation of this difference [40]. We found strong correlations between the EQ-5D-3L and EQ-5D-5L-based SHE results indicating that good estimates can be calculated from one to another in case of lack of available data.

The expected deterioration of health with age measured by the EQ-5D questionnaires was confirmed by the GALI instrument as well (Table 3). More than 80% of the respondents expected to have moderate or severe activity limitation at age 80 and 45% believed to be severely limited at age 90. Moreover, similar to the EQ-5D-based findings (Fig. 1), underestimation of future health for age 70 and over was apparent also with the GALI. According to Eurostat data, some or severe activity limitation (GALI) was present in 34, 47, 65, and 82% in age groups of 55–64, 65–74, 75–84, and 85+ of the Hungarian population, while the sample’s future estimates for ages 60, 70, 80, and 90 were 36, 60, 82, and 86%, respectively [41]. Although statistical and subjective life-expectancy data have to be considered in further analyses, results suggest a gap between the statistical HLY and subjectively expected HLY of the population.

According to participants’ beliefs, health deterioration will be accompanied by a less sharp decrease in happiness in their life, as they indicated in average a value of 6.8 for age 60 and 5.7 for age 90. A worsening of mental health as measured by the WHO-5 was observed in people’s expectations. This can be partly explained by the relatively low current mental well-being of the population. Only 36–52% of the participants indicated to have positive feelings all or most of the time across the five dimensions of the WHO-5. For instance, only 37% reported that daily life was filled with things that interest him/her and 44% indicated that he/she felt calm and relaxed all or most of the time. These findings are congruent with EQ-5D data of the general population in which 35% reported to have moderate or severe problem in the anxiety/depression dimension of the EQ-5D-3L [42]. Expected deterioration of mental well-being for future ages was especially remarkable in the ‘feel cheerful and in good spirits’ and ‘feel active and vigorous’ dimensions (Table 3).

While in the previous SHE studies (based on the EQ-5D-3L), the most important explanatory variables of SHE were age, current EQ-5D-3L status, self-perceived healthy lifestyle, and subjective life expectancy, we found a more heterogeneous picture in our survey [3, 5] (Table 4). Given that age 60 was rather close to the current age of the respondents in a substantial number of cases (Table 1), we focus on SHE for ages 70, 80, and 90. We found that current activity limitation (GALI) and educational level were significant predictors of future EQ-5D-5L expectations at all the three ages (i.e., having no activity limitation and higher educational level induced better expected health scores). Longevity expectations influenced SHE for the oldest ages (80 and 90) (Table 4). Current happiness was an important predictor of future happiness for all ages. In addition, current self-perceived health status and informal care received were also significant determinants (Supplementary Table S2).

Drug expenses are expected to increase with age and the majority of the respondents (49%) expected to spend more than 10% of the net household income on drugs at age 90 (Fig. 2a) This partly reflects the increasing need of drugs in the elderly but probably also a relative decrease of incomes with age. It would be interesting to explore in further studies whether people expect to have limited access to drugs due to personal financial barriers.

According to our best knowledge, this is the first study to assess subjective expectations on care needs and availability of forms of care for the elderly alongside SHE. Our results confirmed that people do count with an increasing need for help with age. Altogether, 2.6, 4.6, 11.2, and 28.6% expected to need 29 h or more of care per week at age 60, 70, 80, and 90, respectively (Table 3). Due to lack of age-specific care-time data, we cannot judge whether it is an overestimation of care needs influenced partly by the underestimation of health status from age 70 and over. A study of Rubovszky 2017 reported an average 26.7 h of care per week in Hungary in a sample, where 41% of the care recipients were aged 60 or older; hence, our participants’ estimates seem to be rather realistic [43].

One of the most important findings of our study is that the majority of the individuals count with the support of family members if they will need long-term help in the future (Fig. 2b). Most of the respondents plan to rely on informal care (77, 72, 53, and 40% for ages 60, 70, 80, and 90, respectively) either in their own home or by moving to the family members’ home to be helped there. More and more individuals expect to move to a care institution with increasing age, although its share does not exceed the informal care expectations at any age (5, 8, 22, and 37%, respectively). The share of respondents considering institutions provided by the state (no co-payment) is higher for all future ages than of those who consider moving to a care institution of own choice but with significant co-payment. Given that about 48% of the samples do not expect to have extra income other than pension after retiring, the low occurrence of co-paid institutionalized care seems to be rational. It would be worthy to investigate more in depth which family members (e.g., partner or child) people expect to help them in their elderly and how much realistic these expectations are considering, for instance, their children’s age and the increasing retirement age in the society. We have not explored whether the low share of care institutions in the expectations reflects availability and access barriers, or rather the preferences of the people.

The expected median age of doing paid work was 65 years. In Hungary, the standard retirement age was 63 years in 2016 and is gradually increasing to 65 in 2022. However, in our sample, even the age group 25–34 expected to retire at age 66 and similar data were found in the others as well. These findings highlight the important fact that the young generation do not count with a substantial increase of retirement age. According to an OECD report, it is suggested to adjust retirement age to increasing life expectancy in Hungary and increasing the statutory retirement age to 70 years in steps from 2029 would fully cover the projected long-term pension spending increase to 2.7% of GDP [44]. Hence, we think that there is a gap between expected and the real retirement age among the young and middle-aged population.

Some limitations of our study have to be mentioned. The part of the Hungarian population that does not use the internet and is not available in online was not represented in our sample. The measurement tools that we used to explore future expectations have been partly successfully used in the previous studies (EQ-5D-3L), but none of them were validated for such purposes. We surveyed subjective expectations for ages 60, 70, 80, and 90, as we aimed to focus on ageing. However, age 60 was probably too low for a substantial proportion of the respondents to be considered as a stage of elderly, as it is under the retirement age in Hungary. On the other hand, for the youngest respondents, age 60 was 30–40 years far in the future; hence, it was important for us to include this age.

In conclusion, our study confirmed that individuals expect a deterioration of health with age but a much slighter decrease of happiness. Educational level and current functioning status (GALI) were significant determinants of SHE, longevity influenced SHE for the oldest ages. Happiness expectations were mainly driven by current happiness. Underestimation of health and overestimation of activity limitations was observed for age 70 and over. Significant increase in care needs with age is expected by the majority and most people think that they will be helped by informal caregivers. Moving to care institutions were forecasted only by a minority and it was more present for age 90; however, its share did not exceed the expected informal care’s share even in this high age. Although retirement age will probably raise substantially in the next decades, even the youngest age groups expect to retire typically at about the current retirement age which is 65 years in Hungary. Our study contributes with valuable inputs from the societal perspective for as inputs for ageing policy, health care capacity and manpower planning, financing, and insurance scheme scenario analyses, projecting the labor force participation, as well as for health economic modelling of ageing.

## Electronic supplementary material

Below is the link to the electronic supplementary material.

Supplementary material 1 (DOCX 44 kb)
