# Supplementary material for: Subjective expectations regarding ageing: a cross-sectional online population survey in Hungary
Source: Eur J Health Econ. 2019 May 20;20(Suppl 1):17–30. doi: 10.1007/s10198-019-01059-w (PMC6544751; doi:10.1007/s10198-019-01059-w)
Supplement: Supplementary file 1 — Supplementary material 1 (DOCX 44 kb) [file 10198_2019_1059_MOESM1_ESM.docx]

**Supplementary file**

**Subjective expectations regarding aging: a cross-sectional online population survey in Hungary**

**The European Journal of Health Economics**

Márta Péntek^1^, Ottó Hajdu^2^, Fanni Rencz^1,3^, Zsuzsanna Beretzky^1,4^, Valentin Brodszky^1^, Petra Baji^1^, Zsombor Zrubka^1,4^, Klára Major^5^, László Gulácsi^1^

**Corresponding author:**

Márta Péntek

Corvinus University of Budapest

Department of Health Economics

H-1093 Budapest, Fővám tér 8.

E-mail: [marta.pentek@uni-corvinus.hu](mailto:marta.pentek@uni-corvinus.hu)

**Supplementary Table S1 Questionnaire used in the survey*: an example how expected health problems at future ages were asked applying the EQ-5D-3L descriptive system.**

| I think at age 60 I will have… *(Please mark your response)* | | | | |
| --- | --- | --- | --- | --- |
| a. | no | some | severe | problems with walking about. |
|  |  |  |  |  |
| b. | no | some | severe | problems with washing or dressing. |
|  |  |  |  |  |
| c. | no | some | severe | problems with performing usual activities. |
|  |  |  |  |  |
| d. | no | some | severe | pain or discomfort. |
|  |  |  |  |  |
| e. | no | some | severe | anxiety or depression. |
|  |  |  |  |  |

*Ages 70, 80 and 90 were asked in a same construct, questions were built similarly with the EQ-5D-5L descriptive system, GALI and WHO-5.

**Supplementary Table S2 Determinants of current and expected happiness for future ages**

| Predictor variables  (X) | Dependent variable: Current happiness | | Dependent variable: expected happiness at age 60 | | Dependent variable: expected happiness at age 70 | | Dependent variable: expected happiness at age 80 | | Dependent variable: expected happiness at age 90 | |
| --- | --- | --- | --- | --- | --- | --- | --- | --- | --- | --- |
|  | R Square 0,454 | | R Square 0,903 | | R Square 0,902 | | R Square 0,785 | | R Square 0,680 | |
|  | Unstandardized Coefficients B | Standardized Coefficients Beta | Unstandardized Coefficients B | Standardized Coefficients Beta | Unstandardized Coefficients B | Standardized Coefficients Beta | Unstandardized Coefficients B | Standardized Coefficients Beta | Unstandardized Coefficients B | Standardized Coefficients Beta |
| (Constant) | -6.758^**(0)^ | … | 10.124^***(0)^ | … | 5.312^**(0)^ | … | 11.117^***(0)^ | … | 13.984^***(0)^ | … |
| Current happiness | … | … | 0.740^***(1)^ | 0.642 | 0.749^***(1)^ | 0.671 | 0.668^***(1)^ | 0.640 | 0.571^***(1)^ | 0.488 |
| EQ-5D-5L | 3.265^**(1)^ | 0.302 | … | … | … | … | … | … | … | … |
| EQ VAS | … | … | … | … | … | … | -0.017^(10)^ | -0.150 | -0.018^(14)^ | -0.147 |
| Activity limitation (GALI | 0.676^(6)^ | 0.171 | … | … | -0.864^**(13)^ | -0.199 | … | … | -0.648^(13)^ | -0.140 |
| Self-perceived health | … | … | … | … | -0.813^***(12)^ | -0.285 | -1.245^***(6)^ | -0.435 | -1.842^***(4)^ | -0.575 |
| Chronic morbidity | 1.661^(8)^ | 0.142 | -2.303^*(6)^ | -0.195 | … | … | -3.530^***(7)^ | -0.291 | -5.604^***(3)^ | -0.408 |
| Age | 0.039^*(3)^ | 0.208 | … | … | 0.066^***(9)^ | 0.275 | … | … | … | … |
| Gender | … | … | -0.886^(3)^ | -0.130 | -0.966^**(5)^ | -0.165 | -1.235^***(3)^ | -0.229 | … | … |
| Educational level | 0.383^*(4)^ | 0.214 | … | … | … | … | … | … | … | … |
| Subjective life-expectancy | … | … | … | … | 0.012^(6)^ | 0.072 | 0.033^**(2)^ | 0.203 | 0.033^*(2)^ | 0.180 |
| Kins’ age at death | … | … | … | … | -0.374^(15)^ | -0.102 | -0.275^(11)^ | -0.078 | -0.514^(12)^ | -0.136 |
| Body Mass Index category | … | … | … | … | … | … | … | … | -0.923^***(6)^ | -0.286 |
| Informal care received, hours/week | -0.013^(7)^ | -0.115 | … | … | 0.028^***(10)^ | 0.226 | 0.028^***(5)^ | 0.225 | 0.042^***(5)^ | 0.309 |
| Informal caregiver | … | … | … | … | … | … | 0.470^(12)^ | 0.087 | 1.085^*(9)^ | 0.184 |
| Marital status: Single | … | … | … | … | -0.722^(7)^ | -0.094 | -1.565^**(4)^ | -0.201 | -1.083^(7)^ | -0.123 |
| Marital status: Divorced | … | … | 2.423^**(4)^ | 0.275 | 1.270^**(4)^ | 0.166 | … | … | 1.011^(10)^ | 0.126 |
| Marital status: Widow | 0.800^(9)^ | 0.126 | … | … | … | … | … | … | … | … |
| Marital status: Has a partner | … | … | … | … | … | … | … | … | … | … |
| Other marital status | … | … | … | … | … | … | … | … | … | … |
| Net household monthly income | … | … | … | … | … | … | … | … | 0.185^(11)^ | 0.139 |
| Self-perceived healthy lifestyle | … | … | -2.284^***(2)^ | -0.473 | -1.358^***(2)^ | -0.286 | -0.698^*(8)^ | -0.168 | … | … |
| Smoking status | 0.214^(10)^ | 0.100 | … | … | 0.318^*(11)^ | 0.134 | -0.200^(13)^ | -0.090 | … | … |
| Sport activities | 0.883^(5)^ | 0.174 | … | … | … | … | … | … | … | … |
| Alcohol consumption | … | … | … | … | … | … | … | … | … | … |
| Employment status: Works part-time | … | … | … | … | … | … | -3.428^**(9)^ | -0.166 | … | … |
| Employment status: Retired | … | … | … | … | … | … | … | … | … | … |
| Employment status: Disability pensioner | … | … | -0.757^(7)^ | -0.108 | -0.964^**(8)^ | -0.150 | … | … | … | … |
| Employment status: Student | … | … | … | … | … | … | … | … | … | … |
| Employment status: Unemployed (seeking job) | … | … | -3.441^**(5)^ | -0.210 | -1.486^(3)^ | -0.104 | … | … | … | … |
| Employment status: Unemployed (not seeking job) | 6.746^***(2)^ | 0.338 | … | … | 1.629^(14)^ | 0.082 | … | … | 3.598^(8)^ | 0.154 |
| Employment status: Housewife/husband | … | … | … | … | … | … | … | … | … | … |
| Other employment status | … | … | … | … | … | … | … | … | … | … |

Notes: Significance level (p-value): *between 0.1 and 0.05; **between 0.05 and 0.01; *** <0.01; No*: p-value >0.1.

(#)Stepwise entry number.

Explanatory variables indicated as ‘…’ in the Table were not involved into the model by the regression model-specification. For dummies (marital and employment status): the first category (marital status: married; employment status: works in a full time job) was used as reference. Maximum points of corrected R squared define the actual list of predictors.

Coding: GALI: 1-severely limited, 2-limited but not severely, 3-not limited; Gender: 0-female, 1-male; Educational level: 1-no primary education, 10-university; Chronic morbidity: 1-yes, 2-no; Kins’ age at death: 1- 55-64 years, 2: 65-74, 3- 75-84, 4-85-94, 5- 95 years or over; Body Mass Index Category:1-underweigth, 2-normal, 3-overweight, 4- obese; Self-perceived health: 1-very good, 2-good, 3-fair, 4-bad, 5-very bad; Informal caregiver experience: 1-yes, currently; 2-yes in the past but not currently, 3-no; Net household monthly income: 1- 0-159 EUR/month, 11- 1593 EUR/month or over; Self-perceived healthy lifestyle: 1-healthier than others, 2- comparable to others, 3- less healthy than others; Smoking status: 1- current smoker, 2-quitted smoking within a yea2, 3-quitted smoking more than a year ago, 4-never smoked; Alcohol consumption: 1: every day or nearly every day, …, 9: never only a few times in my life; Dummy: 1-yes, 2-no.

**Supplementary Table S3 Expected forms of care for age 80 (N=990)**

| **Variables** |  | **Own home, helped by volunteer family members** | **Moving to the home of close relatives and being helped there** | **Own home, helped by a carer paid by myself** | **Institution provided by the healthcare/social system with no co-payment** | **Institution by own choice with significant co-payment** | **Other** |
| --- | --- | --- | --- | --- | --- | --- | --- |
| **N, %** |  |  |  |  |  |  |  |
| Total |  | 375 (37.9%) | 147 (14.8%) | 183 (18.5%) | 126 (12.7%) | 88  (8.9%) | 71  (7.2%) |
| Gender |  |  |  |  |  |  |  |
| female |  | **184 (49.1%)*** | **96 (65.3%)*** | 107 (58.5%) | 72 (57.1%) | 58 (65.9%) | **26 (36.6%)*** |
| male |  | **191 (50.9%)*** | **51 (34.7%)*** | 76 (41.5%) | 54 (42.9%) | 30 (34.1%) | **45 (63.4%)*** |
| Educational level |  |  |  |  |  |  |  |
| primary |  | 103 (27.5%) | 54 (36.7%) | **43 (23.5%)*** | **51 (40.5%)*** | 19 (21.6%) | 28 (39.4%) |
| secondary |  | 165 (44.0%) | 54 (36.7% | **69 (37.7%)*** | **55 (43.7%)*** | 44 (50.0%) | 32 (45.1%) |
| tertiary |  | 107 (28.5%) | 39 (26.5%) | **71 (38.8%)*** | **20 (15.9%)*** | 25 (28.4%) | 11 (15.5%) |
| Employment |  |  |  |  |  |  |  |
| employed |  | **175 (46.7%)*** | 86 (58.5%) | 102 (55.7%) | 64 (50.8%) | 46 (52.3%) | 43 (60.6%) |
| not employed |  | **200 (53.3%)*** | 61 (41.5%) | 81 (44.3%) | 62 (49.2%) | 42 (47.7%) | 28 (39.4%) |
| **Median; mean (SD)** |  |  |  |  |  |  |  |
| Age, years** |  | 55.0;  53.4 (15.9) | 50.0;  48.5 (15.0) | 53.0;  51.0 (15.0) | 48.0;  48.4 (12.9) | 53.0;  47.8 (15.6) | 45.0;  46.1 (11.6) |
| Current EQ-5D-5L index** |  | 0.937;  0,888 (0.154) | 0.922;  0.882 (0.126) | 0.922;  0.815 (0.198) | 0.879;  0.815 (0.198) | 0.906;  0.845 (0.178) | 0.922;  0.846 (0.219) |
| Current happiness (0-10)** |  | 8.0;  7.2 (2.3) | 7.0;  7.0 (2.1) | 7.0;  6.6 (2.2) | 6.0;  6.1 (2.4) | 7.0;  6.5 (2.6) | 6.0;  5.7 (2.7) |
| Expected EQ-5D-5L index for age 80** |  | 0.740;  0.679 (0.304) | 0.640;  0.547 (0.336) | 0.630;  0.541 (0.334) | 0.488;  0.364 (0.403) | 0.593;  0.446 (0.391) | 0.593;  0.428 (0.509) |
| Expected happiness for age 80** |  | 7.0;  6.9 (2.4) | 7.0;  6.6 (2.3) | 6.0;  6.0 (2.4) | 5.0;  5.0 (2.9) | 6.0;  5.9 (2.9) | 5.0;  4.7 (3.6) |

*Difference between subgroups is significant p<0,01 (Chi squared)

**Age, current EQ-5D-5L index and happiness score, expected EQ-5D-5L and expected happiness score for age 80 years were significantly different (p=0.000) across types of care. (Kruskal-Wallis test)
